# Gold(I) Complexes of *N*-Heterocyclic Carbene Ligands Containing Benzimidazole: Synthesis and Antimicrobial Activity

**DOI:** 10.3390/molecules15042203

**Published:** 2010-03-29

**Authors:** İlknur Özdemir, Nazan Temelli, Selami Günal, Serpil Demir

**Affiliations:** 1 Department of Chemistry, Faculty of Science and Art, İnönü University, 44280 Malatya, Turkey; E-Mails: nzntemelli@hotmail.com (N.T.); 2 Department of Microbiology, Faculty of Medicine, İnönü University, 44280 Malatya, Turkey; E-Mail: selamig@inonu.edu.tr (S.G.)

**Keywords:** gold, *N*-heterocyclic carbene, metallopharmaceutical agent, antimicrobial, benzimidazol-2-ylidene

## Abstract

Gold(I) *N*-heterocyclic carbene (NHC) complexes were obtained in good yields from the corresponding silver complexes by treatment with [AuCl(PPh_3_)] following the commonly used silver carbene transfer route. The silver complexes were synthesized from the benzimidazolium halide salts by the *in situ* reactions with Ag_2_O in dichloromethane as a solvent at room temperature. All gold complexes have been characterized by ^1^H-NMR, ^13^C-NMR and IR spectroscopy and elemental analysis. Au-NHC complexes were evaluated for their *in vitro* antimicrobial activity against a variety of Gram-positive and Gram-negative bacteria and fungal species.

## 1. Introduction 

Medicinal inorganic chemistry is a discipline of growing significance in both therapeutic and diagnostic medicine. The history and basic concepts of medicinal inorganic chemistry have been recently reviewed [1,2]. The application of silver and its salts in the treatment of burn wounds is thus of special interest [3], and has prompted an upsurge in research on the synthesis of Ag(I) complexes with projected antibacterial applications [4,5]. In 1985 auranofin, an orally bioavailable, monomeric gold(I) phosphine drug was introduced for rheumatoid arthritis [6]. Neutral [Au(NHC)L] type compounds, analogues of [Au(PEt_3_)Cl] and auranofin, have also been discussed. The biomedical applications of metal complexes based on *N*-heterocyclic carbenes [7,8] are just beginning to unfold, despite such complexes being phenomenally successful in homogeneous catalysis [9]. Ghosh and co-workers reported the design and utility of NHC-metal complexes such as Pd, Ag, and Au as metallopharmaceuticals [5,10]. Recently, Ag-NHC complexes that showed significant antimicrobial activity were reported by Youngs and Lin [7,11,12]. Au NHC complexes, although known for decades [13], have only recently made greater impact on NHC chemistry due to the potential applications in medicinal chemistry spanning antiarthritic [14], to antitumor [15] to antimicrobial activities [16]. 

The activation strategies used for the preparation of NHC-metal complexes can be divided into: (i) cleavage of the C=C bond of electron rich alkenes [17]; (ii) generation of a free carbene by deprotonation of the corresponding imidazolium precursor with a strong base [18]; (iii) transmetallation of a deprotonated azole followed by protonation or alkylation of the gold azolyl compounds [19]; (iv) *in situ* deprotonation of an imidazolium salt with a weak base [20], (v) transmetallation from a silver-NHC complex prepared by the direct reaction of an imidazolium precursor with Ag_2_O [21]. Among these methods, the Ag-carbene transfer route comprises over 70% of the published results; the free carbene route constitutes around 20%. 

In this work, we describe the preparation, characterization and antimicrobial activity of the [Au(NHC)Cl] type complexes. These complexes were characterized with different spectral techniques like ^1^H-NMR, ^13^C-NMR and IR spectroscopy and elemental analysis. All gold complexes showed antibacterial activity against the tested Gram (+)/(-) and fungal strains.

## 2. Results and Discussion

### 2.1. Synthesis of gold carbene complexes ***1***–***3***

**Scheme 1:**
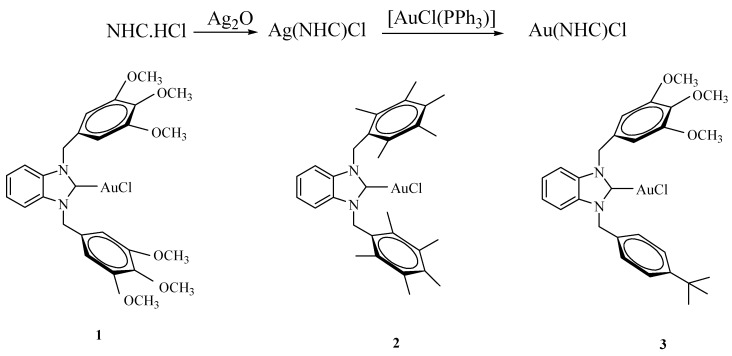
Gold(I) *N*-Heterocyclic Carbene Complexes.

The symmetrical and unsymmetrical benzimidazolium salts were obtained as reported in the literature [22]. The [Au(NHC)Cl] type complexes were synthesized according to Ag(I)-NHC transfer method [23], whereby the corresponding benzimidazolium chloride was treated with silver oxide in CH_2_Cl_2_, and the resulting mixture was allowed to react with [AuCl(PPh_3_)]. The advantages of this method are that no pregeneration of the free carbene is necessary. This reaction can be performed in air with no decrease in yield, and no decomposition to metallic gold. This route entails the formation of a silver carbene complex *in situ* by the treatment of the corresponding benzimidazolium salt with Ag_2_O and subsequent transmetalation with [AuCl(PPh_3_)] to generate the analogous Au(I)-NHC complex in good yields (Scheme 1).

The new products **1–3** were obtained as white crystalline complexes in 93, 89 and 83% yield, respectively. The gold complexes have been characterized by analytical and spectroscopic techniques. The conversion of a benzimidazolium salt into the corresponding Au-NHC complex is characterized by the disappearance of the signal due to the acidic benzimidazolium (NC*H*N) proton in the ^1^H-NMR spectrum and, in the ^13^C-NMR spectrum, by the appearance of a signal due to the C2 (carbene) of the benzimidazol-2-ylidene units at 191.4, 191.9 and 191.1 ppm, respectively, for **1–3**. Complexes can be obtained after recrystallization from dichloromethane/hexane as crystalline materials, readily soluble in most common polar organic solvents, but insoluble in hexane or diethyl ether. The carbene compounds are thermally stable at room temperature. The IR data for gold-carbene complexes clearly indicate the presence of the –C-N-group with a υ_(CN)_ vibration between 1398 and 1406 cm^−1^, as expected.

### 2.2. Antimicrobial properties of gold(I)NHC complexes

The antibacterial activities of the gold complexes are reported in terms of the minimum inhibitory concentration (MIC) values, which are defined as the lowest concentration of an antimicrobial compound that visibly inhibits the growth of the bacteria after an overnight incubation. The antimicrobial activities were evaluated against *Staphylococcus aureus, Enterococcus faecalis*, *Escherichia coli*, *Pseudomonas aeruginosa*, *Candida albicans* and *Candida tropicalis* and were compared with ampicillin, ciprofloxacin, and fluconazole, all used to treat general bacterial infections. The results are summarized in Table 1. 

The observed differences in activity of the series of Au(I)-NHC inhibitors corroborate the hypothesis that a subtle combination of electronics and sterics in the carbene bound to the gold ion leads to dramatic differences in inhibition. Especially the complexes have shown antibacterial activity to different extents, depending on the nature of the ligand. Compound **2** is more active than compounds **1** and **3** against the *C. albians* and *C. tropicalis* fungal strains (MICs 12.5 µg/mL). Incorporation of same groups as substituents on the *N*-atom (**1** and **3**) resulted in similar activity against all bacterial strains. At the same time compounds **1** and **3** inhibited the growth of Gram positive *S. aureus and E. faecalis* bacterial strains, but did not show good effect against Gram negative bacteria and fungal strains (MICs 200–400 µg/mL). In the case of incorporation of a pentamethylbenzyl group on the aromatic ring (compound **2**) only antifungal activity was exhibited. From the data obtained in this work, it is suggested that the substituents on the *N*-atom may play a crucial role in the antimicrobial activity. 

## 3. Experimental 

### 3.1. General

All preparative reactions were carried out under argon in flame-dried glassware using standard Schlenk techniques. The solvents used were purified by distillation over the drying agents indicated and were transferred under Ar: Et_2_O (Na/K alloy), CH_2_Cl_2_ (P_4_O_10_), hexane (Na). Melting points were determined in glass capillaries under air with an Electrothermal-9200 melting point apparatus. FT-IR spectra were recorded as KBr pellets in the range 400–4000 cm^−1^ on an ATI UNICAM 1000 spectrometer. ^1^H-NMR and ^13^C-NMR spectra were recorded in CDCl_3_ with tetramethylsilane as an internal reference using a Varian AS 400 Merkur spectrometer operating at 400 MHz (^1^H) or 100 MHz (^13^C). Elemental analyses were carried out by analytical service of TÜBİTAK with a Carlo Erba Strumentaziona Model 1106 apparatus. 

### 3.2. General method for the preparation of gold NHC complexes

A mixture of Ag_2_O (0.25 mmol) and benzimidazolium salt (0.5 mmol) was stirred in CH_2_Cl_2_ (30 mL) at ambient temperature for 6 h, then [AuCl(PPh_3_)] (0.5 mmol) was added and the mixture was stirred for 4 h. The mixture was filtered through Celite to remove the precipated silver chloride. The solvent was removed under vacuum and the residue was redissolved in CH_2_Cl_2_ (5 mL) and hexane (10 mL) was added. At that time phosphine that was removed from the starting Au complex was filtered and after that the resulting white solid was collected, washed with hexane and recrystallized from dichloromethane/hexane at room temperature.

*Chloro-[1,3-bis(3,4,5-trimethoxybenzyl)benzimidazole-2-ylidene]gold (I)* (**1**). Yield: 0.661 g; 93%, m.p. 207–208 °C; υ_(CN)_ = 1402 cm ^−1^; ^1^H-NMR δ: 7.36 (s, 4 H, NC_6_*H*_4_N), 6.49 (s, 4 H, CH_2_C_6_*H*_2_(OCH_3_)_3_-3,4,5), 5.79 (s, 4H, C*H*_2_C_6_H_2_(OCH_3_)_3_-3,4,5), 3.78 and 3.66 (s, 18 H, CH_2_C_6_H_2_(OC*H*_3_)_3_-3,4,5); ^13^C-NMR δ: 191.4 (*C*_carbene_), 112.4, 125.0 128.9 and 133.9 (N*C*_6_H_4_N), 104.3, 131.0, 137.9 and 153.6 (CH_2_*C*_6_H_2_(OCH_3_)_3_-3,4,5), 60.7 and 56.3 (CH_2_C_6_H_2_(O*C*H_3_)_3_-3,4,5), 52.6 (*C*H_2_C_6_H_2_(OCH_3_)_3_-3,4,5); Anal. Calcd. for C_27_H_30_N_2_O_6_AuCl: C, 45.61; H, 4.25; N, 3.94%; found: C, 45.65; H, 4.21; N, 3.98. 

*Chloro-[1,3-bis(2,3,4,5,6-pentamethylbenzyl)benzimidazole-2-ylidene]gold (I)* (**2**). Yield: 0.597 g; 89%, m.p. 229–230 °C; υ_(CN)_ = 1406 cm ^−1^; ^1^H-NMR (CDCl_3_) δ: 7.14–7.10 (m, 2 H, NC_6_*H*_4_N), 6.91–6.87 (m, 2 H, NC_6_*H*_4_N), 5.96 (s, 4 H, C*H*_2_C_6_(CH_3_)_5_-2,3,4,5,6), 2.37, 2.24 and 2.22 (s, 30 H, CH_2_C_6_(C*H*_3_)_5_-2,3,4,5,6); ^13^C-NMR δ: 191.9 (*C*_carbene_), 112.7, 127.6, 129.0 and 134.1 (N*C*_6_H_4_N), 124.8, 133.5, 133.6 and 136.4 (CH_2_*C*_6_(CH_3_)_5_-2,3,4,5,6), 51.1 (*C*H_2_C_6_(CH_3_)_5_-2,3,4,5,6), 17.7, 17.3 and 17.0 (CH_2_C_6_(*C*H_3_)_5_-2,3,4,5,6); Anal. Calcd. for C_31_H_38_N_2_AuCl: C, 55.48; H, 5.71; N, 4.17%; found: C, 55.53; H, 5.67; N, 4.14. 

*Chloro-[1-(3,4,5-trimethoxybenzyl)-3(ter-butylbenzyl)benzimidazole-2-ylidene]gold (I)* (**3**). Yield: 0.562 g; 83%, m.p. 253–254 °C; υ_(CN)_ = 1398 cm ^−1^; ^1^H-NMR δ: 7.48–7.35 (m, 4 H, NC_6_*H*_4_N), 7.26 and 7.17 (d, *J =* 8.4 Hz, 4 H, CH_2_C_6_*H*_4_C(CH_3_)_3_-*p*), 6.45 (s, 2 H, CH_2_C_6_*H*_2_(OCH_3_)_3_-3,4,5), 5.85 and 5.82 (s, 4 H, C*H*_2_C_6_H_2_(OCH_3_)_3_-3,4,5 and C*H*_2_C_6_H_4_C(CH_3_)_3_-*p*), 3.77 and 3.63 (s, 9 H, CH_2_C_6_H_2_(OC*H*_3_)_3_-3,4,5), 1.22 (s, 9 H, CH_2_C_6_H_4_C(C*H*_3_)_3_-*p*); ^13^C-NMR δ: 191.1 (*C*_carbene_), 103.7, 112.2, 112.3, 124.9, 125.8, 127.0, 131.1, 132.5, 133.6, 133.8, 137.6, 151.4 and 153.6 (CH_2_*C*_6_H_2_(OCH_3_)_3_-3,4,5, CH_2_*C*_6_H_4_C(CH_3_)_3_-*p* and N*C*_6_H_4_N), 60.7 and 56.1 (CH_2_C_6_H_2_(O*C*H_3_)_3_-3,4,5), 52.5 and 52.3 (*C*H_2_C_6_H_2_(OCH_3_)_3_-3,4,5) and *C*H_2_C_6_H_4_C(CH_3_)_3_-*p*), 34.5 (CH_2_C_6_H_4_*C*(CH_3_)_3_-*p*), 31.2 (CH_2_C_6_H_4_C(*C*H_3_)_3_-*p*); Anal. Calcd. for C_28_H_32_N_2_O_3_AuCl: C, 49.68; H, 4.76; N, 4.14%; found: C, 49.73; H, 4.72; N, 4.19. 

### 3.3. Antimicrobial activities of silver NHC complexes

Antimicrobial activities of the Ag (I) complexes with *N*-heterocyclic carbene ligands were determined by using the agar dilution procedure recommended by the Clinical and Laboratory Standards Institute [24,25]. Minimal inhibitory concentrations for each compound were investigated against standard bacterial strains. *Staphylococcus aureus* ATCC 29213, *Enterococcus faecalis* ATCC 29212, *Escherichia coli* ATCC 25922, *Pseudomonas aeruginosa* ATCC 27853 were obtained from American Type Culture Collection (Rockville, MD.) and the fungal strains *Candida albicans* and *Candida tropicalis* were obtained from the Department of Microbiology, Faculty of Medicine, Ege University (Turkey). Bacterial strains were subcultured on Muller Hinton Broth (HiMedia Laboratories Pvt. Ltd. Mumbai-India) and fungal strains were also on RPMI 1640 Broth (Sigma-Aldrich Chemie GmbH Taufkirchen, Germany). Their turbidities matched that of a McFarland no. 0.5 turbidity standard [26]. The stock solution of all compounds was prepared in dimethyl sulfoxide (DMSO). All of the dilutions were done with distilled water. The concentrations of the tested compounds were 800, 400, 200, 100, 50, 25, and 12.5 µg/mL. Ampicillin, ciprofloxacin were used as antibacterial standard drugs, while fluconazole were used as antifungal standard drugs whose minimum inhibitory concentration (MIC) values are provided. A loopful (0.01 mL) of the standardised inoculums of the bacteria and yeasts (10^6^ CFUs/mL) was spread over the surface of agar plates. All the inoculated plates were incubated at 35 °C and results were evaluated after 16–20 h of incubation for bacteria and 48 h for yeasts. The lowest concentration of the compounds that prevented visible growth was considered to be the minimal inhibitory concentration (MIC).

## 4. Conclusions 

In summary, we have presented the synthesis and characterization of some novel benzimidazole-2-ylidene Au(I) complexes. The antibacterial activity of the Au(NHC)Cl complexes was tested *in vitro* against various microbial strains. The gold complex **2** showed only antifungal activity, while **1** and **3** inhibited the growth of Gram positive bacteria better than **2**. It was found that antimicrobial activity of the gold(I) carbene complexes against different kinds of bacteria and fungi varies with the nature of the ligand. Research in our laboratory is underway to explore the catalytic activities of all these gold complexes. Also detailed investigations focusing on new Au and Ag-NHC complexes as metallopharmaceutical agent and other biomedical applications are ongoing. 

## Figures and Tables

**Table 1:** Minimum inhibitory concentration (μg/mL) of silver NHC complexes tested against bacterials.

Bacteria	1	2	3	Ampicillin	Ciprofloxacin	Fluconazole
***E. coli***	400	400	200	3.12	1.56	-
***S. aureus***	12.5	200	12.5	3.12	0.39	-
***E. faecalis***	12.5	200	12.5	1.56	0.78	-
***P. aerug***	400	800	400	-	3.12	-
***C. albicans***	200	12.5	200	-	-	3.12
***C. tropicalis***	200	12.5	200	-	-	3.12
